# Altered functional connectivity is associated with Repeatable Battery for the Assessment of Neuropsychological Status across the dementia spectrum

**DOI:** 10.1017/S135561772610191X

**Published:** 2026-04-01

**Authors:** Jace B. King, Molly B.D. Prigge, Vincent Koppelmans, John M. Hoffman, Kevin Duff

**Affiliations:** 1 Department of Radiology and Imaging Sciences, Spencer Fox Eccles School of Medicine, University of Utah, Salt Lake City, UT, USA; 2 Department of Psychiatry, Spencer Fox Eccles School of Medicine, University of Utah, Salt Lake City, UT, USA; 3 Layton Aging & Alzheimer’s Disease Research Center, Department of Neurology, Oregon Health & Science University, Portland, OR, USA

**Keywords:** fMRI, brain, cognitive aging, cognitive dysfunction, Alzheimer’s disease, neuropsychological tests

## Abstract

**Objective:**

The quest for non-invasive and cost-effective biomarkers for mild cognitive impairment (MCI) and Alzheimer’s disease (AD) has led to growing interest in resting-state functional magnetic resonance imaging (MRI). This study examined associations between whole-brain functional connectivity measures and cognitive performance across a spectrum of cognitive aging.

**Method:**

A total of 108 older adults (mean age 74.1 ± 5.7 years), comprised of cognitively intact individuals, participants with amnestic MCI, and those with mild dementia due to probable AD, underwent high-resolution structural MRI and resting-state functional MRI scans and cognitive testing with the Repeatable Battery for the Assessment of Neuropsychological Status (RBANS). Functional connectivity values were derived from a 17-network brain parcellation. Correlations were established between network connectivity values and RBANS Index scores.

**Results:**

Analyses revealed that lower RBANS Attention Index and Total Scale scores were significantly associated with increased connectivity between the ventral attention, central executive network, and limbic and default mode networks. Lower RBANS total scores were also associated with functional connectivity strength between the dorsal default mode networks and lateral frontoparietal regions of the central executive network, with increased connectivity observed across the dementia spectrum (Intact-MCI-AD).

**Conclusions:**

These findings suggest that aberrant and potentially compensatory increases in functional connectivity may be linked to cognitive decline, supporting the utility of resting-state functional MRI as a promising biomarker for MCI and AD.

## Statement of Research Significance

**Research Questions(s) or Topic(s):** This study analyzed the relationship between brain network activity and memory and cognitive skills in older adults across a range of cognitive aging and dementia. **Main Findings:** The study revealed that weaker cognitive abilities relate to stronger connections both between and within certain brain networks. Individuals with lower cognitive performance exhibited stronger connections among brain areas associated with attention, memory, and decision-making. This pattern became more pronounced in participants with more severe cognitive issues, such as Alzheimer’s disease, compared to those with milder forms of cognitive impairment. **Study Contributions:** These findings suggest that the brain may attempt to compensate for cognitive decline by strengthening connections between specific networks. This study supports the notion that resting-state brain scans could aid in detecting early signs of mild cognitive impairment and Alzheimer’s disease-related dementia, thereby making diagnosis easier and more accessible.

## Introduction

The identification of biomarkers in mild cognitive impairment (MCI) and Alzheimer’s disease (AD) has the potential to advance the diagnosis, tracking, and treatment of these conditions. Although several biomarkers are available (e.g., *β*-amyloid plaque via positron emission tomography (PET), tau in cerebrospinal fluid via lumbar puncture, brain metabolism via fluoro-2-deoxyglucose PET imaging, and apolipoprotein ϵ4 carrier status and other plasma markers via blood draw), these diagnostic procedures can be expensive and/or invasive and expose potential participants to unnecessary risks (Chang et al., 2017; Holland et al., 2012; Hua et al., 2016; Kennedy et al., 2014; Lorenzi et al., 2010; Macklin et al., 2013; Sevigny et al., 2016; Yu et al., 2014).

Resting-state functional MRI (fMRI) utilizes naturally occurring fluctuations in low-frequency oscillations of blood oxygen level-dependent (BOLD) contrast between brain regions while the brain is not engaged in a particular cognitive task (i.e., at rest). Functional connectivity is a quantitative measure of synchronous brain activity used to identify large-scale resting-state functional networks (Biswal et al., 1995; Buckner et al., 2011; Fox et al., 2006; Greicius et al., 2003; Yeo et al., 2011). This measure of brain activity is altered in patients with MCI and AD. Specifically, between- and within-network connectivity in multiple canonical networks (e.g., default, salience, central executive, limbic) seems particularly affected (Agosta et al., 2012; Badhwar et al., 2017; Bai et al., 2009; Binnewijzend et al., 2012; Brier et al., 2012; Greicius et al., 2004; Jalilianhasanpour et al., 2019; Koch et al., 2012; Lehmann et al., 2015; Lehmann et al., 2013; Petrella et al., 2011; Ranasinghe et al., 2014; Sorg et al., 2007; Zhang et al., 2010). However, across studies, poorer cognitive performance has been associated with both reduced (‘hypo-’) connectivity and increased (‘hyper-’) connectivity within and between large-scale networks (Badhwar et al., 2017), leaving the directionality of functional connectivity findings in these groups uncertain. For resting-state functional connectivity to serve as a potential biomarker for identifying MCI and AD, it is essential to establish a relationship between functional connectivity and established markers of cognitive change.

The Repeatable Battery for the Assessment of Neuropsychological Status (RBANS) is a brief neuropsychological screening measure that assesses language, attention, visuospatial/constructional faculties, and immediate and delayed memory (Randolph et al., 1998). Considerable research has validated the RBANS in evaluating cognitive function in cognitively intact older individuals (Cooley et al., 2015; Duff & Ramezani, 2015; Phillips et al., 2015; Thaler et al., 2015), MCI (Clark et al., 2010; Duff et al., 2010; Hobson et al., 2010; Karantzoulis et al., 2013; O’Mahar et al., 2012), and AD (Burton et al., 2015; Duff et al., 2008; Enright et al., 2015; Heyanka et al., 2015; McDermott & DeFilippis, 2010; Morgan et al., 2010; Schmitt et al., 2010). Moreover, RBANS performance has been associated with existing MCI and AD brain biomarkers such as hippocampal atrophy (Duff et al., 2023; England et al., 2014; Euler et al., 2023; Ottoy et al., 2019; Paul et al., 2011; Suhrie et al., 2021), hypometabolism on fluoro-2-deoxyglucose (FDG) PET (Frings et al., 2016; Jagust et al., 2010), cerebral *β*-amyloid deposition (Duff et al., 2013; Euler et al., 2023; Hammers et al., 2017; Ottoy et al., 2019; Suhrie et al., 2021; Teng et al., 2019), and APOE ϵ4 carrier status (Duff et al., 2023; Suhrie et al., 2021).

A growing body of literature has demonstrated associations between resting-state functional connectivity between canonical networks and cognitive performance across various tasks and assessments (Contreras et al., 2017; Gour et al., 2014; Li et al., 2016; Lin et al., 2018), suggesting functional connectivity-based biomarkers may generalize across instruments that assess comparable domains. However, to our knowledge, no study has specifically explored whether patterns of resting-state functional connectivity are linked to performance on the RBANS and its index scores. Establishing RBANS-specific functional connectivity correlations in older adults remains important since this brief battery is well-tolerated by this population, has appropriate normative data, has alternate forms for tracking cognition over time, and has been validated in older adults who are cognitively unimpaired or have MCI or AD (Randolph et al., 1998). It is also widely used in clinical practice (Rabin et al., 2016) and clinical trials (Papp et al., 2022). As such, understanding the associations of functional connectivity and performance on the RBANS appears to have practical applications.

The goal of the current study was to examine the relationship between the RBANS Index and Total scores and resting-state functional connectivity in participants identified as cognitively intact, MCI, or AD. Whole-brain resting-state functional connectivity of large-scale functional networks, including the visual, somatomotor, attention, salience, central executive, and default mode networks (Yeo et al., 2011), was examined. Given the equivocal pattern of directional predictions for MCI and AD relative to cognitively intact older adults (hypo- versus hyper-connectivity), no such directional predictions were made. Instead, diagnostic status was conceptualized as a position along a dementia-spectrum continuum, and group membership was coded as an ordinal index of clinical severity. It was hypothesized that functional connectivity within and between networks of interest would demonstrate stronger associations with cognitive performance as dementia-spectrum status increased (ranging from cognitively intact to MCI to AD), reflecting progressive disruption of network organization as it relates to cognition.

## Methods

### Participants

This study was approved by the Institutional Review Board at the University of Utah and conducted in compliance with the Helsinki Declaration. Participants were recruited from a cognitive disorders clinic or through the community and received remuneration for their voluntary participation. Data were collected between 2018 and 2022. All study participants provided written informed assent or consent prior to study participation. Participants were required to be 65 or older and have a knowledgeable collateral source available to speak to their cognitive and daily functioning. Their mean age was 74.1 ± 5.7 years, and mean education was 16.1 ± 2.5 years. Most were Caucasian (98.2%), and 58.3% were female. A total of 108 participants were included in the study, and using the classification battery described below, 47 participants were classified as cognitively intact, 31 as amnestic MCI (single or multidomain), and 30 as mild dementia due to probable AD. Detailed demographics of the participants are provided in Table 1.

**Table 1.** Demographics and cognitive and imaging outcomes

Variable	Intact (*N* = 47)	MCI (*N* = 31)	AD (*N* = 30)	Total (*N* = 108)		
					*χ* ^2^	*p*-value
Sex (% Female)	61.7	58.1	53.3	58.3	0.53	.768
Race (% Caucasian)	100.0	93.5	100.0	98.2	5.06	.281
					*F*	*p*-value
Age (years)	72.1 ± 4.7 [65–91]	74.0 ± 5.2 [67–87]	77.4 ± 6.4 [66–89]	74.1 ± 5.7 [65–91]	8.76	** ***<.001
Education (years)	16.9 ± 1.9 [12–20]	15.0 ± 2.6 [12–20]	16.0 ± 2.5 [12–20]	16.1 ± 2.4 [12–20]	6.49	*.002
WRAT-4 reading	110.5 ± 7.3 [88–126]	107.9 ± 9.7 [88–134]	110.1 ± 8.8 [96–126]	109.6 ± 8.4 [88–134]	0.93	.398
MMSE	29.5 ± 0.8 [27–30]	26.4 ± 1.9 [22–29]	23.1 ± 2.7 [18–27]	26.8 ± 3.2 [18–30]	110.89	* ** ***<.001
CDR global score	0.0 ± 0.1 [0–0.50]	0.5 ± 0.0 [0.5–0.5]	0.8 ± 0.2 [0.5–1.0]	0.4 ± 0.4 [0–1]	357.73	* ** ***<.001
WMS-R	12.1 ± 3.9 [5–20]	2.8 ± 2.8 [0–12]	1.2 ± 1.4 [0–5]	6.4 ± 5.9 [0–20]	146.28	* **<.001
Mean head motion (mm)	0.2 ± 0.1 [0.09–0.43]	0.2 ± 0.1 [0.1–0.4]	0.3 ± 0.1 [0.1–0.5]	0.2 ± 0.1 [0.1–0.5]	2.87	.061
Total brain volume (cm^3^)	1005.0 ± 99.3 [813.8–1,171.5]	937.3 ± 98.2 [746.8–1,132.4]	947.0 ± 96.6 [723.2–1,131.8]	969.5 ± 102.3 [723.2–1,171.5]	5.42	* **.005
RBANS index						
Immediate memory	107.2 ± 13.4 [69–136]	76.6 ± 13.0 [53–103]	67.4 ± 14.6 [44–100]	87.4 ± 22.3 [44–136]	91.50	* ** ***<.001
Visuospatial/Constructional	107.4 ± 14.4 [64–131]	95.3 ± 16.6 [62–131]	92.7 ± 20.4 [56–121]	99.8 ± 18.0 [56–131]	8.56	* **<.001
Language	103.6 ± 11.1 [85–130]	89.3 ± 9.9 [60–108]	85.5 ± 11.6 [51–110]	94.5 ± 13.6 [51–130]	29.93	* **<.001
Attention	108.0 ± 13.6 [79–138]	95.3 ± 13.2 [75–122]	89.8 ± 16.4 [49–125]	99.3 ± 16.2 [49–138]	16.56	* **<.001
Delayed memory	110.0 ± 12.4 [78–137]	66.7 ± 18.2 [40–98]	52.5 ± 11.7 [40–82]	81.6 ± 29.2 [40–137]	175.66	* ** ***<.001
RBANS total scale	110.4 ± 13.7 [81–140]	80.6 ± 10.5 [62–101]	70.6 ± 14.5 [40–97]	90.8 ± 21.9 [40–140]	97.36	* ** ***<.001

Data are presented as means ± standard deviations [min–max] unless otherwise noted. For cognitive test scores, MMSE and WMS-*R* are raw scores, and WRAT-4 and RBANS are standard scores (*M* = 100, SD = 15). Between-group differences in sex and race distributions were calculated using Pearson’s Chi-Square. *Post hoc* Bonferroni test (*p* < .05).

*Intact vs. MCI.

**Intact vs. AD.

***AD vs. MCI.

Intact = cognitively intact; AD = Alzheimer’s disease; CDR = Clinical Dementia Rating Scale; MCI = mild cognitive impairment; MMSE = Mini-Mental Status Examination; RBANS = Repeatable Battery for the Assessment of Neuropsychological Status; WMS-R = Wechsler Memory Scale-Revised Logical Memory II Paragraph A; WRAT-4 = Wide Range Achievement Test 4^th^ edition.

Exclusion criteria included enrollment in a clinical drug trial related to anti-amyloid agents, medical comorbidities likely to affect cognition (e.g., neurological conditions, current severe depression, substance abuse, and major psychiatric conditions), or the inability to complete neuroimaging visits (MRI or PET). Additional exclusion criteria included elevated depression as indicated by a score of greater than 5 on the 15-item Geriatric Depression Scale (Yesavage et al., 1982) and moderate or severe dementia as indicated by a global Clinical Dementia Rating score of 2 or greater or a Mini-Mental Status Examination (Folstein et al., 1975) score of less than 20.

### Group assignment and measures

Baseline testing was used to assign participants to a participant group (cognitively intact, MCI, or AD) according to the Alzheimer’s Disease Neuroimaging Initiative (ADNI2) classification battery, which included the Mini-Mental Status Examination (Folstein et al., 1975), the Clinical Dementia Rating Scale (Morris, 1993), and the Wechsler Memory Scale-Revised (Wechsler, 1987) Logical Memory II Paragraph A. The AD group consisted of individuals with mild dementia due to probable AD. Mean premorbid intellectual functioning was assessed using the Reading subtest of the Wide Range Achievement Test – version 4 (Wilkinson & Robertson, 2006). Additional baseline neuropsychological testing included the RBANS, a concise neuropsychological screening tool utilized to evaluate various cognitive domains, including attention, language, visuospatial/constructional skills, as well as both immediate and delayed memory (Randolph et al., 1998). RBANS Index scores use age-adjusted normative comparisons to produce standard scores (*M* = 100, SD = 15), where higher scores reflect better cognition. All RBANS Index scores, as well as Total Scale scores, were included in the analysis.

### Imaging data

Approximately 7 days (7.0 ± 0.8 days) after the baseline testing visit, imaging data were acquired at the Utah Center for Advanced Imaging Research using a Siemens Prisma 3 Tesla MRI scanner (80 mT/m gradients) with a 64-channel head coil. Structural images consisted of a MP2RAGE sequence (TR = 5000 ms; TE = 2.93 ms; flip angles = 4° and 5°, FOV = 256 mm, 176 sagittal slices, resolution = 1.0 mm isotropic; TI1 = 700 ms; TI2 = 2030 ms). Resting-state functional images were acquired using a multi-band EPI sequence (TR = 737 ms; TE = 33.2 ms; flip angle = 52°; FOV = 208 mm; 72 axial slices; resolution = 2.0 mm isotropic; multi-band acceleration factor = 8; partial Fourier = 6/8; bandwidth = 2004 Hz/Px). Two resting-state acquisitions of 1220 volumes (time series duration = 15 min 09 s each; one left-to-right and one right-to-left) were acquired along with pulse and respiration waveform data. Before resting-state scan acquisition, participants were instructed to rest but remain awake with their eyes open while letting their thoughts wander. No visual fixation stimuli were used.

### Imaging analysis

Structural data were processed using FreeSurfer (v6.0.0), which is documented and freely available for download (http://surfer.nmr.mgh.harvard.edu/). For technical details related to FreeSurfer data analysis, see (Fischl & Dale, 2000; Fischl et al., 2002, 2004). Gray matter atrophy can influence functional connectivity results, which is commonly observed in aging populations (Binnewijzend et al., 2012; Damoiseaux et al., 2012; Hafkemeijer et al., 2015; Passamonti et al., 2019). As such, the FreeSurfer output variable, BrainSegNotVent, was extracted for each study participant as a measure of total brain volume and used as a covariate in functional connectivity analyses. Resting-state fMRI data were analyzed using SPM12 software (Wellcome Trust, London) for MATLAB (MathWorks, Natick MA). The processing pipeline involved motion correction of resting-state fMRI images (realignment), coregistration of the realigned functional images to the structural image (MP2RAGE), segmentation of the MP2RAGE, and normalization of the coregistered MP2RAGE and functional images to an MNI152 template. Motion parameters from the realignment step were stored for later volume censoring. All further analyses of resting-state were conducted in MNI space. Phase-shifted soft tissue correction (PSTCor) (Anderson et al., 2011) was then used to regress six detrended participant motion parameters: soft tissues of the face and calvarium, degraded white matter, degraded cerebrospinal fluid, and physiological waveforms (Birn et al., 2008). Volumes before and after root-mean-square head motion greater than 0.5 mm were censored using motion parameters provided by the processing pipeline (Power et al., 2012). There were no significant differences in mean head motion between groups (see Table 1). Resting-state analysis was conducted on 116 participants. Participants who had less than 50% of the original 1220 volumes (for each scan) remaining following motion censoring were removed. After the removal of eight participants due to motion censoring, 108 participants were included in the present analysis.

After preprocessing, resting-state functional MRI data were analyzed using a 17-network brain parcellation scheme (Yeo et al., 2011), as previously described (King & Anderson, 2018). This parcellation includes visual, somatomotor, dorsal attention, salience, limbic, executive control, and default mode networks. This parcellation is a finer-grained parcellation of the more widely used 7-network parcellation (Yeo et al., 2011). The 17-network parcellation includes subdivisions of the larger networks, allowing for within-network analyses in addition to between-network. In addition, the finer-grained parcellation minimizes the risk of averaging time courses across large, potentially heterogeneous regions. Time series data from each of the 17 distributed brain networks were extracted for analysis, with each network treated as a single region of interest (ROI). We quantified functional connectivity as the Pearson correlation between regional BOLD time series. This method is supported by extensive evidence showing that correlation-based functional connectivity captures key large-scale systems and task networks (Smith et al., 2009), is reproducible and reliable with modern denoising techniques (Noble et al., 2019), and enables robust prediction and individual identification (Finn et al., 2015). Additionally, at the macroscale, resting-state BOLD dynamics are well approximated by linear models, making linear association a logical and interpretable measure (Nozari et al., 2024). For each subject, a matrix consisting of Fisher-transformed Pearson correlation coefficients representing functional connectivity for 17 × 17 networks was created by averaging across both resting-state scans. Each matrix contains the 136 unique inter-network edges.

### Statistical analysis

A one-way analysis of variance (ANOVA) was used to detect between-group differences for continuous measures with post hoc Bonferroni tests to correct for multiple comparisons. A *χ* ^2^ test was used to compare frequency distributions of sex and race across groups. Non-imaging-based statistical analyses were performed using SPSS (version 27.0; SPSS, Chicago, IL, USA).

The primary analysis evaluated RBANS Index (Attention, Language, Immediate Memory, Delayed Memory, Visuospatial/Constructional) and Total Scale scores and functional connectivity across the dementia spectrum (cognitively intact, MCI, and AD treated as ordinal variables). Analyses were conducted in MATLAB (MathWorks, Natick MA). For each cognitive measure and each of the 136 edges, partial correlation values were estimated between functional connectivity and cognition, controlling for mean head motion, age, sex, and total brain volume. These covariates are all well-established sources of variance in resting-state functional connectivity and cognition, which could otherwise confound the relationship between them. Aging changes large-scale network connections and is associated with cognitive decline (Andrews-Hanna et al., 2007; Ferreira & Busatto, 2013; Geerligs et al., 2014). Sex differences also influence functional connectivity patterns and cognition, and these are robust enough that functional connectivity alone can classify sex (Satterthwaite et al., 2014; Weis et al., 2019; Zhang et al., 2018). Even submillimeter head motion biases functional connectivity estimates, typically inflating local correlations and decreasing long-range correlations, so motion must be modeled at the individual level even if group means do not differ (Ciric et al., 2017; Power et al., 2012; Satterthwaite et al., 2013; Van Dijk et al., 2012). Total brain volume was included to account for global atrophy effects that are closely linked to both cognitive impairment and AD stage. Structural MRI markers of neurodegeneration, including total brain and cortical volume loss, are key features of AD pathology and reflect clinical severity (Fox et al., 1999; Jack et al., 2018; Whitwell et al., 2007). Resting-state studies in AD explicitly associate gray matter atrophy with functional connectivity, demonstrating that connectivity differences are not solely due to tissue loss (Binnewijzend et al., 2012; Damoiseaux et al., 2012; Hafkemeijer et al., 2015; Passamonti et al., 2019). Notably, we did not include education in our model, despite significant group differences, because education can be viewed as a proxy of cognitive reserve, which may moderate how pathology translates into measured cognition (Bennett et al., 2003; Roe et al., 2008; Stern, 2012). Furthermore, adjusting for this potential mediator can introduce overadjustment bias (Schisterman et al., 2009). Partial correlations (*r*) were computed with Pearson correlation on residuals. Results include *r* as the effect size, the two-sided p-value, and a 95% confidence interval (CI) using Fisher’s *z* transformation with standard error 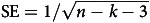 , where *n* is the complete-case sample size and *k* is the number of covariates (here, *k* = 4). Correction for multiple comparisons across the 136 edges was completed using the Benjamini-Hochberg procedure at *q* < .05. Uncorrected results (*p* < .05) are provided in the form of figures and tables in the Supplementary Material for all RBANS Index and Total Scale findings.

*Group differences.* To test functional connectivity differences between diagnostic groups, linear models for each edge were fit with functional connectivity as the dependent variable and group plus covariates (mean head motion, age, sex total brain volume) as predictors. Analyses included unique, unordered pairwise contrasts ordered by clinical severity (AD vs intact, AD vs MCI, and MCI vs intact), with the group factor re-levelled so the less severe level served as the reference. The t-statistic with degrees of freedom, two-sided p-value, FDR-adjusted *q*-value (within-contrast across 136 edges), and a standardized effect size, 
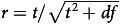
 , with confidence intervals, are reported for each significant edge. Full results (*p* < .05, uncorrected) are provided in the Supplementary Material for all edges.

## Results

No differences in sex, race, mean head motion, or mean premorbid intellectual functioning were found between the three diagnostic groups (see Table 1 for details). The AD group was older than the intact and MCI groups, and the MCI group had less education than the intact group (see Table 1). All RBANS Index and Total Scale scores were significantly lower in AD and MCI participants compared to cognitively intact individuals. The AD group performed significantly worse than the MCI group on Immediate and Delayed Memory Indexes and Total Scale scores (see Table 1). Total brain volumes were significantly smaller in those with MCI and AD compared to the intact participants. No significant difference in brain volume was found between the MCI and AD groups.

**Figure 1.**
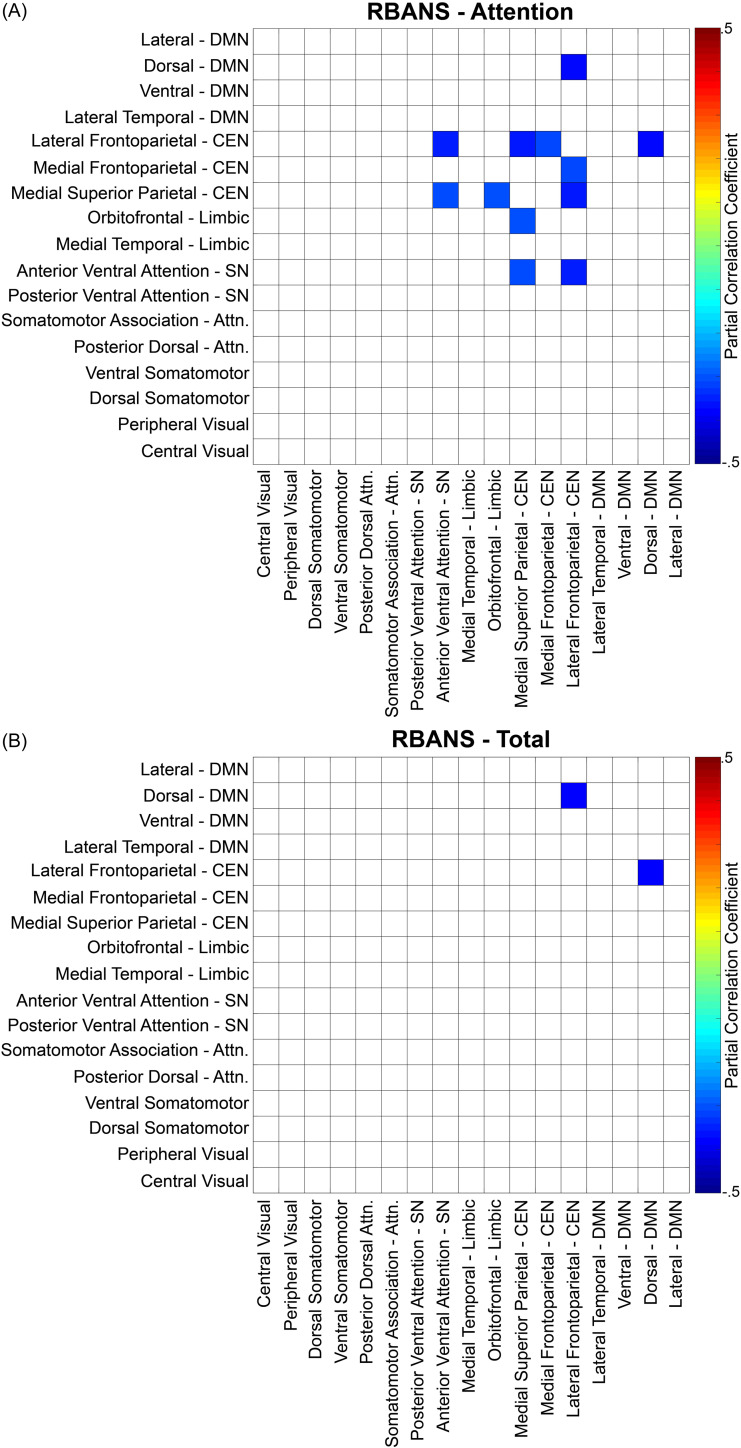
Across diagnostic groups (Intact-MCI-AD), RBANS Attention Index (A) and Total (B) Scores were negatively associated with resting-state functional connectivity values across a 17-network parcellation (qFDR < .05). Data represent partial correlation coefficients controlling for the effects of age, sex, mean head motion, and total gray matter volume. RBANS = Repeatable Battery for the Assessment of Neuropsychological Status; Attn. = Attention; CEN = Central Executive Network; DMN = Default Mode Network; SN = Salience Network.

**Figure 2.**
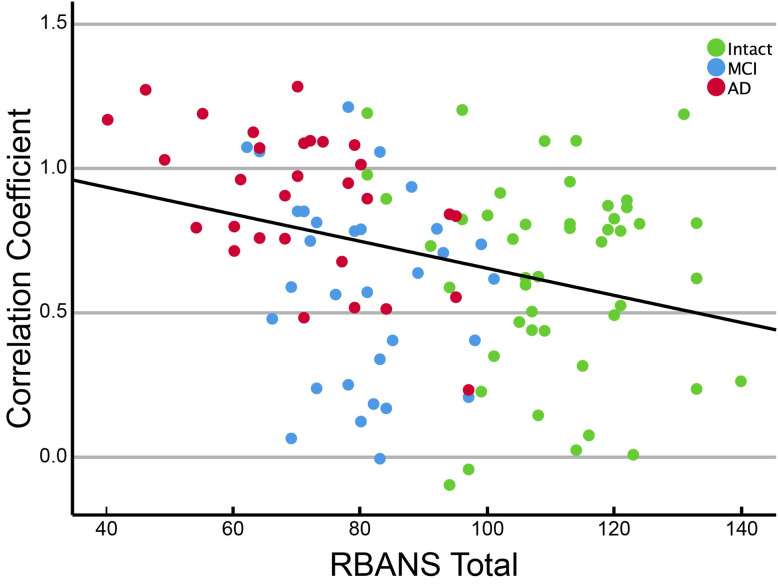
Simple scatter plot representing the relationship between RBANS Total Scores (x-axis) and functional connectivity Fisher-transformed correlation coefficient values between the lateral frontoparietal (central executive) and dorsal default mode networks (*y*-axis).

In the full study sample, RBANS Attention Index demonstrated a significant negative correlation with functional connectivity, suggesting that lower (worse) RBANS Index scores are associated with stronger functional connectivity between regions. This pattern of associations was found within subcomponents of the central executive network and between central executive and salience, default mode, and limbic subcomponent networks. Within the central executive network, RBANS Attention scores were negatively associated with functional connectivity between the lateral frontoparietal and medial superior parietal (*r* = −.35, [95% CI = −.51, −.17], *p* < .001, *qFDR* = .013) and medial frontoparietal (*r* = −.31, [95% CI = −.47, −.12], *p* = .002, *qFDR* = .046) regions. RBANS Attention scores were also negatively associated with functional connectivity between subcomponents of the salience and central executive networks (anterior ventral attention with lateral frontoparietal (*r* = −.35, [95% CI = −.51, −.17], *p* < .001, *qFDR* = .013) and medial superior parietal (*r* = −.30, [95% CI = −.47, −.12], *p* = .002, *qFDR* = .046). This pattern was also found between subcomponents of the central executive and default mode networks (lateral frontoparietal and dorsal default mode, *r* = −.37, [95% CI = −.52, −.19], *p* < .001, *qFDR* = .013), and between subcomponents of the central executive and orbitofrontal limbic networks (medial superior parietal and orbitofrontal limbic, *r* = −.30, [95% CI = −.46, −.11], *p* = .002, *qFDR* = .047) (see Figure 1A). RBANS Total Scale scores demonstrated a similar pattern between the central executive (lateral frontoparietal) and the dorsal default mode networks (*r* = −.38, [95% CI = −.53, −.20], *p* < .001, *qFDR* = .010; see Figures 1B and 2). Functional connectivity was not significantly correlated with any other RBANS index scores when correcting for multiple comparisons. However, uncorrected results demonstrated a similar pattern of primarily lower RBANS Index scores with increasing functional connectivity in central executive and default mode networks (see Supplementary Material Figures 1–6 and associated Tables).

To assist in interpreting dimensional effects in relation to diagnostic categories, an exploratory post hoc analysis of between-group differences was conducted. For each network connection, a linear regression model was fitted, with diagnosis (cognitively intact, MCI, AD) as a categorical predictor and the same covariates as in the primary model. From these models, adjusted pairwise contrasts were derived (cognitively intact vs MCI, cognitively intact vs AD, and MCI vs AD). No significant differences were found between cognitively intact and MCI participants. A significant increase in connectivity was observed in the AD group compared to MCI between the default mode and central executive networks (dorsal default mode and lateral frontoparietal, *t*(101) = 4.38, *p* < .001, *qFDR* = .004, *r* = .40 [95% CI = .22, .55], and dorsal default mode and medial frontoparietal, *t*(101) = 3.36, *p* = .001, *qFDR* = .049, *r* = .32 [95% CI = .13, .48]) and between the central executive and salience networks (lateral frontoparietal and anterior ventral attention, *t*(101) = 4.03, *p* < .001, *qFDR* = .007, *r* = .37 [95% CI = .19, .53]). A similar pattern of increased functional connectivity between the central executive and default mode and salience networks emerged when comparing the AD group to cognitively intact individuals, but without involvement of the medial frontoparietal subcomponent (dorsal default mode and lateral frontoparietal, *t*(101) = 4.55, *p* < .001, *qFDR* = .002, *r* = .41 [95% CI = .25, .56]; lateral frontoparietal and anterior ventral attention, *t*(101) = 3.68, *p* < .001, *qFDR* = .026, *r* = .34 [95% CI = .16, .50]) (see Supplementary Material Figures 7–9 and associated Table).

## Discussion

This study explored the relationship between cognitive functioning, as assessed with the RBANS, and resting-state functional connectivity across 17 large-scale intrinsic functional brain networks in older adults with intact cognitive function, amnestic MCI, and mild AD. Across the dementia spectrum, multilinear regression analyses revealed an association between lower (poorer) RBANS Attention Index scores with increased functional connectivity in default mode, central executive, limbic, and ventral attention (salience) networks. RBANS Total Scale scores were also negatively correlated with functional connectivity strength between the lateral frontoparietal (central executive) and the dorsal default mode networks. These functional brain networks are critical for numerous cognitive functions, including memory, attention, emotional processing, and cognitive control. Taken together, these findings suggest that disruptions in functional connectivity within and between these networks may be associated with cognitive decline in individuals along the dementia spectrum.

An exploratory analysis of between-group functional connectivity revealed no significant differences between cognitively intact and MCI participants. Such a null finding has also been reported in the literature, with several resting-state fMRI studies observing no significant differences in static functional connectivity between controls and individuals with amnestic MCI (Eyler et al., 2019; Malotaux et al., 2022; Weiler et al., 2014). Our study also found a significant increase in connectivity in the AD group compared to MCI and cognitively intact participants between the default mode and central executive networks. Additionally, increased connectivity between the ventral attention (salience) and central executive networks was observed in AD compared to MCI. Atypical (hypo- or hyper-) functional connectivity in both the central executive and the default mode networks has been widely reported in individuals with AD (Binnewijzend et al., 2012; Damoiseaux et al., 2012; Gour et al., 2014; Greicius et al., 2004; Grieder et al., 2018; Jones et al., 2017; Jones et al., 2016; Wiepert et al., 2017). In a longitudinal study, Damoiseaux and colleagues also reported increased functional connectivity in the anterior and ventral default mode networks and decreased connectivity in the posterior default mode network in AD compared to controls at baseline (Damoiseaux et al., 2012). At a follow-up scan 2–4 years later, all three default mode subnetworks demonstrated decreased functional connectivity in AD compared to controls, suggesting an initial compensatory mechanism in early AD. Gour and colleagues (Gour et al., 2014) also found mixed directional functional connectivity in AD compared to controls: increased in the dorsolateral prefrontal cortex and in the left antero-medial temporal network but decreased in default mode and left and right attention networks. Like the present study, Damoiseaux and Gour also controlled for gray matter volume (Damoiseaux et al., 2012; Gour et al., 2014).

In the early stages of cognitive decline, decreases in functional connectivity may represent an initial response to neuronal dysfunction or as the direct result of neuronal damage. Later increases in functional connectivity (late MCI or early AD) may indicate an attempted compensatory response intended to maintain cognitive functioning. For example, increased connectivity within the default mode network has been associated with semantic memory deficits in patients with MCI, suggesting that these increases may be maladaptive or compensatory (Gardini et al., 2015). However, this compensation may come at the expense of increased metabolic demand and inefficiency and may ultimately fail as dementia progresses to AD. Notably, aberrant hyperconnectivity within the default mode network is characteristic of AD, potentially signaling the propagation of amyloid pathology that undermines network integrity (Jones et al., 2017; Jones et al., 2016; Roemer-Cassiano et al., 2025). The cascading network failure model of AD suggests a progressive systems-level breakdown of large-scale functional networks, rather than solely focal pathology. Using resting-state fMRI across the dementia spectrum, Jones and colleagues demonstrated that disruption starts within posterior default mode network subsystems, with early increases in connectivity within and between default mode network hubs and other control networks. This is interpreted as metabolically costly compensatory hyperconnectivity, followed by a progression to widespread hypoconnectivity and network disconnection as the functional networks further decompensate (Jones et al., 2016). Multimodal PET studies support this model by showing that functional network failure is closely linked to tau pathology, with amyloid serving as a partial mediator of the relationship between network failure and tau deposition along connected pathways (Jones et al., 2017).

These disturbances in healthy network connections likely impede efficient cognitive processing, which may influence RBANS scores. Attention deficits are particularly associated with disruptions in the ventral attention (salience) network, which helps switch between internally and externally focused tasks, and the central executive network, which supports working memory and decision-making (Menon, 2011). Increased connectivity in these networks could indicate inefficiency in managing attention-demanding tasks. Moreover, the observed trend (Intact-MCI-AD) suggests that functional connectivity changes may progress simultaneously with cognitive and functional decline. This supports the notion that increased connectivity, initially compensatory, may become maladaptive as dementia develops.

The RBANS Attention Index score assesses basic attention processes and processing speed. Increased functional connectivity in default mode, central executive, limbic, and ventral attention (salience) networks correlated with lower RBANS Attention Index scores. The default mode, central executive, and ventral attention (salience) networks are all central to attention processes and processing speed (Hellyer et al., 2014; Menon, 2011; Staffaroni et al., 2018). Effective coordination between these networks, particularly salience-driven switching between default mode and executive control states, facilitates rapid detection, evaluation, and response to stimuli, supporting focused and flexible cognitive performance (Chand et al., 2017; Cole, 2024; Seeley et al., 2007; Sridharan et al., 2008). While, to our knowledge, this is the first study to correlate resting-state fMRI functional connectivity within and between resting-state networks with RBANS scores across the cognitive aging spectrum, alternative measures assessing attention and cognitive abilities have been investigated in the literature. For example, in a sample of early- and late-onset AD participants, Gour and colleagues found positive associations between measures of executive function, including Rey’s Complex Figure Copy performance score and the Wechsler Memory Scale – Third Edition forward and backward visuospatial span score, and functional connectivity in the dorsolateral prefrontal network (Gour et al., 2014). They also found a positive correlation between visual recognition memory, using delayed recognition performance scores from the Delayed Matching to Sample Task 48, and the antero-medial temporal network (Gour et al., 2014). Contreras et al. found a significant positive relationship between verbal episodic memory and increased connectivity between the frontoparietal and default mode networks (Contreras et al., 2017) in a group of older adults. In another study that included older adults with MCI and AD, significant negative correlations were found between Mini-Mental Status Examination, Montreal Cognitive Assessment, and California Verbal Learning Test scores and functional connectivity strength between the left dorsal frontal (central executive) and right lateral temporal cortices (default mode network) (Li et al., 2016). Finally, using the 11-item Alzheimer’s Disease Assessment Scale – Cognitive Subscale score as a measure of overall cognitive function, Lin et al. found that resting-state functional connectivity, measured using both Pearson’s *r* correlation and accordance/discordance measures, could significantly predict individual differences in a heterogeneous sample that included cognitively normal controls, individuals with MCI, and individuals with AD (Lin et al., 2018). Overall, these findings suggest that functional connectivity patterns in specific brain networks are linked to cognitive performance across aging and dementia; however, the directionality of these associations remains equivocal and uncertain.

Inconsistencies in directionality across studies often arise from whether functional connectivity is within- or between-network, whether global signal regression was performed (as it can invert or induce anticorrelations), and from motion-handling and other denoising choices that influence brain–behavior relationships (Ciric et al., 2017; Murphy et al., 2009; Murphy & Fox, 2017; Saad et al., 2012; Siegel et al., 2016; Smith et al., 2009). These factors vary widely across studies, leading to different results regarding the directionality of connectivity findings even when examining similar constructs. Additionally, data acquisition parameters, like repetition time, phase encoding direction, and scan duration, may also impact connectivity outcomes. When interpreting conflicting results across studies, it is important to keep these various factors in mind. In the present study, networks were predefined and then evaluated to determine whether coupling among these systems relates to cognition across cognitively intact, MCI, and AD participants. The results indicated a consistent pattern: higher connectivity was associated with poorer cognitive performance. Although stronger coupling is often seen as “integration,” it can also indicate maladaptive synchronization, expanded co-activation of typically distinct systems, or over-recruitment of control circuits, which becomes inefficient as pathology progresses. In this context, “increased connectivity” represents a loss of functional specificity, or noisier, less selective communication, rather than a beneficial enhancement of information flow. The consistent direction of this association across different diagnostic groups suggests that it is unlikely to be a chance finding tied to a single disease stage.

There are limitations to the present study that deserve mention. First, the sample size per group was relatively small, and there were unequal sample sizes. Although correlations between RBANS scores and functional connectivity were not analyzed by group, the uneven distribution across groups could affect these factors. However, Figure 2 demonstrates that both connectivity values and RBANS span the entire distribution without gaps, suggesting that unequal group sampling has not negatively affected estimates across groups. Nevertheless, a larger sample size with matched subgroups should be obtained in future studies to reproduce these findings. Second, functional connectivity was defined using the Pearson correlation coefficient between and within canonical brain networks, which may not fully capture BOLD interdependencies, and alternate methods may have yielded different results (Mohanty et al., 2020). Third, gray matter atrophy may influence functional connectivity. While this study attempted to account for this variable by using gray matter volumes as a covariate in regression analyses, the relationship between gray matter atrophy and functional connectivity may be regionally specific. Future studies should employ more precise measures to control for regional atrophy. Fourth, as the parent study was focused on AD, recruitment purposefully focused on individuals presenting with memory concerns and who had amnestic cognitive profiles, both in the MCI and mild dementia states. Consistent with this, both the MCI and AD groups showed their greatest impairment on measures of episodic learning and delayed recall. However, compared to estimates of premorbid intellect (i.e., WRAT-4 Reading), they also averaged more than one standard deviation decrements in language, which would be consistent with AD. As such, the current findings do not necessarily capture the neuropsychology-connectivity associations in non-amnestic subtypes of MCI or non-AD dementias. Despite these limitations, the current study shows that an individual’s unique pattern of whole-brain functional connectivity contains essential information related to cognitive function associated with MCI and AD. These findings suggest that functional connectivity between intrinsic functional brain networks derived from resting-state fMRI may act as an emerging biomarker for AD that holds promise for early diagnosis and treatment monitoring.

## Supporting information

King et al. supplementary material 1 King et al. supplementary material

King et al. supplementary material 2 King et al. supplementary material

## Supplementary material

The supplementary material for this article can be found at https://doi.org/10.1017/S135561772610191X.
