# Supplementary material for: Altered functional connectivity is associated with Repeatable Battery for the Assessment of Neuropsychological Status across the dementia spectrum
Source: J Int Neuropsychol Soc. 2026 Apr 1:1–12. Online ahead of print. doi: 10.1017/S135561772610191X (PMC13061410; doi:10.1017/S135561772610191X)

Altered Functional Connectivity is Associated with Repeatable Battery for the Assessment of Neuropsychological Status Across the Dementia Spectrum

Supplementary Material

Supplementary Material Figures 1-6. Across diagnostic groups (cognitively intact, MCI, and AD treated as ordinal variables), RBANS Index and Total Scores were negatively (primarily) associated with resting-state functional connectivity across a 17-network parcellation ($p < .05$, uncorrected). Data represent partial correlation coefficients controlling for the effects of age, sex, mean head motion, and total gray matter volume.

Supplementary Material Figures 7-9. Results from a post hoc analysis comparing functional connectivity between diagnostic groups. Linear models for each edge were fit with functional connectivity as the dependent variable and group plus covariates (mean head motion, age, sex total brain volume) as predictors. Analyses included unique, unordered pairwise contrasts ordered by clinical severity (AD vs intact, AD vs MCI, and MCI vs intact), with the group factor re-leveled so the less severe level served as the reference. Data represent a standardized effect size (r) with $p < .05$. *qFDR* results have been indicated with *.

Supplementary Material Tables (Separate Excel File) contain results ($p < .05$, uncorrected) from all analyses. *qFDR* corrected results have been **bolded**.

Abbreviations: AD = Alzheimer's disease; Attn. = Attention; CEN = Central Executive Network; DMN = Default Mode Network; MCI = Mild Cognitive Impairment; RBANS = Repeatable Battery for the Assessment of Neuropsychological Status; SN = Salience Network.

Supplementary Material Figure 1. Correlation Between RBANS Immediate Memory Index Scores and Functional Connectivity ($p < .05$, uncorrected).

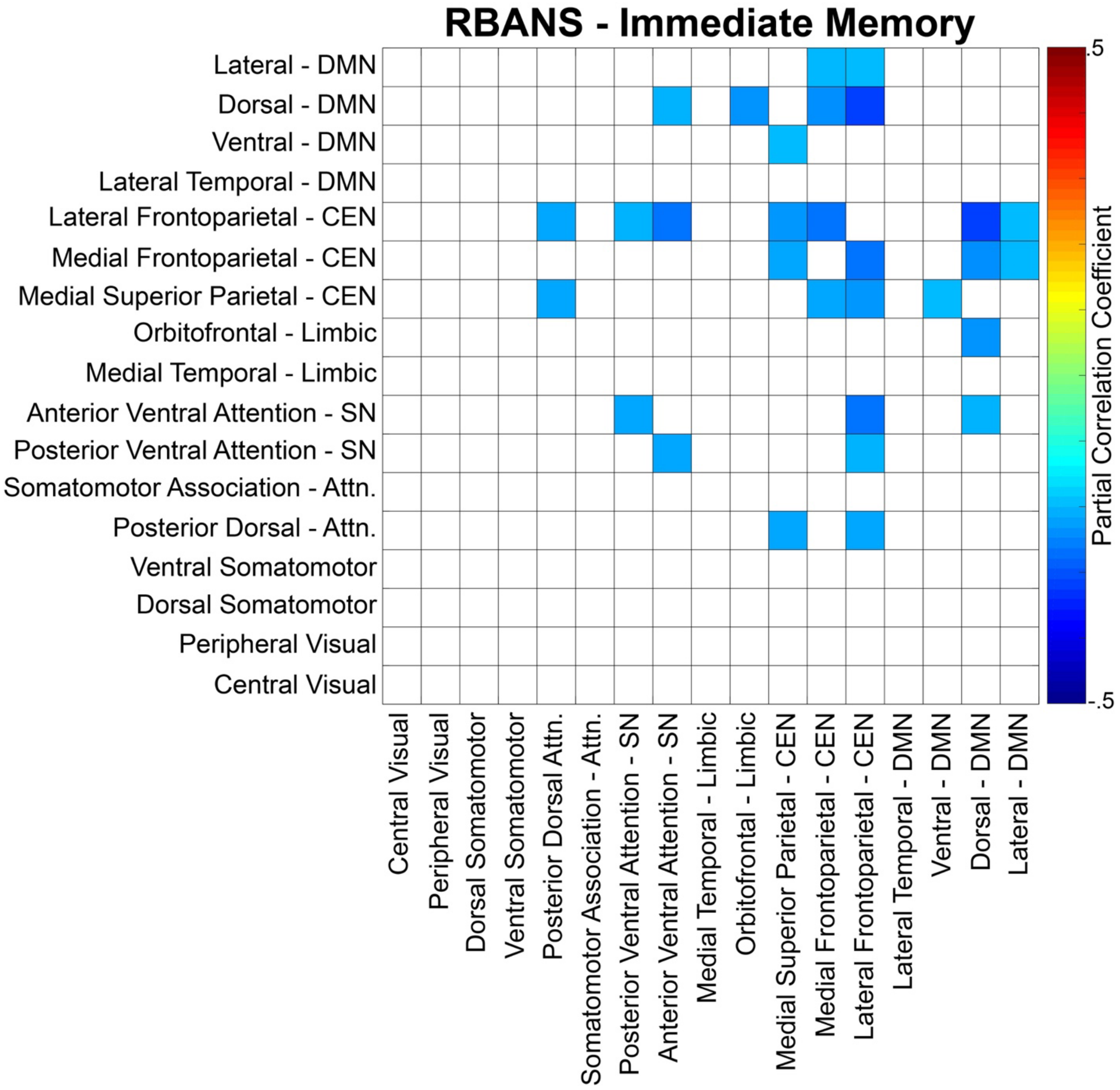

Supplementary Material Figure 2. Correlation Between RBANS Visuospatial/Constructional Index Scores and Functional Connectivity ($p < .05$, uncorrected).

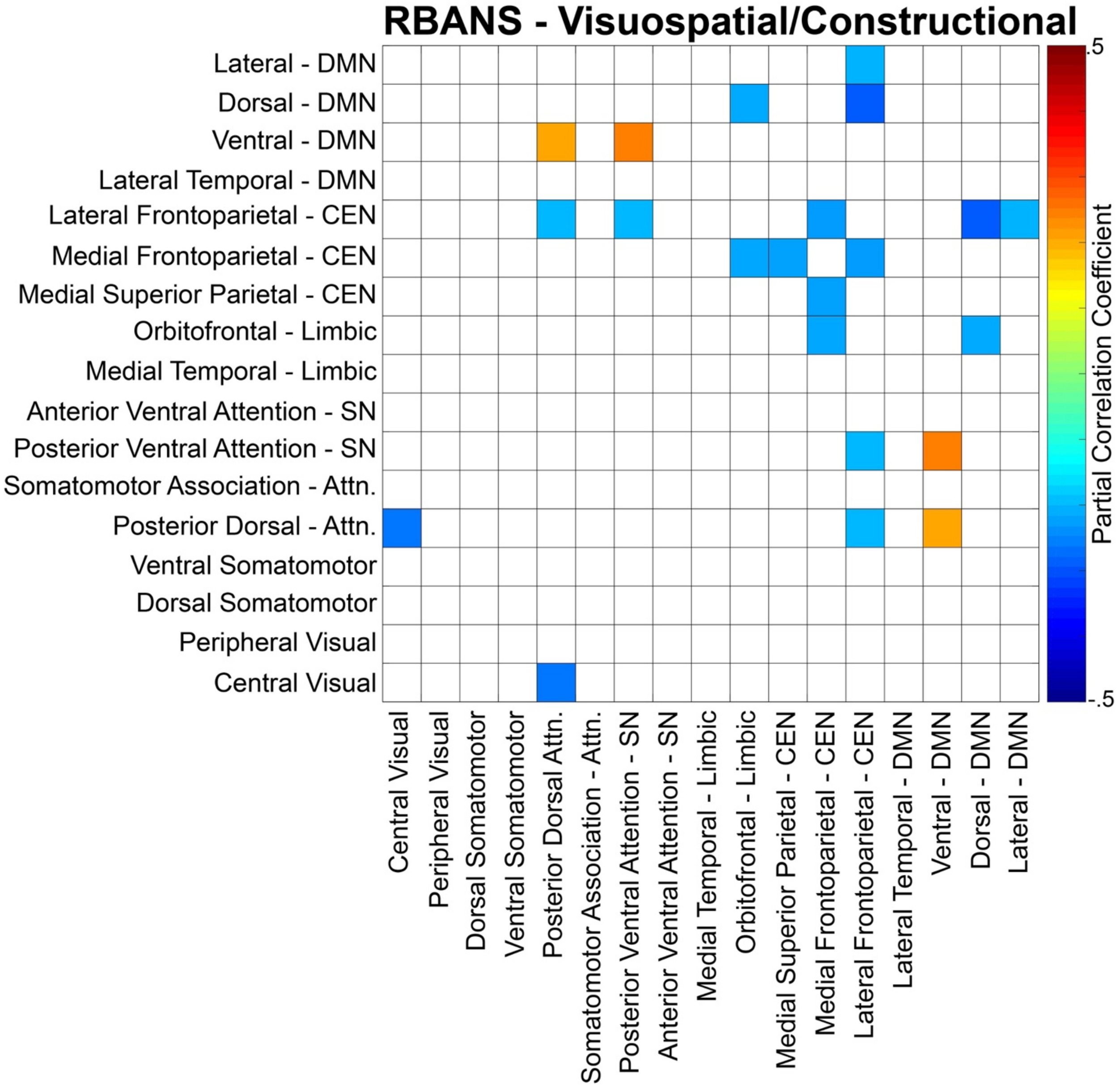

Supplementary Material Figure 3. Correlation Between RBANS Language Index Scores and Functional Connectivity ($p < .05$, uncorrected).

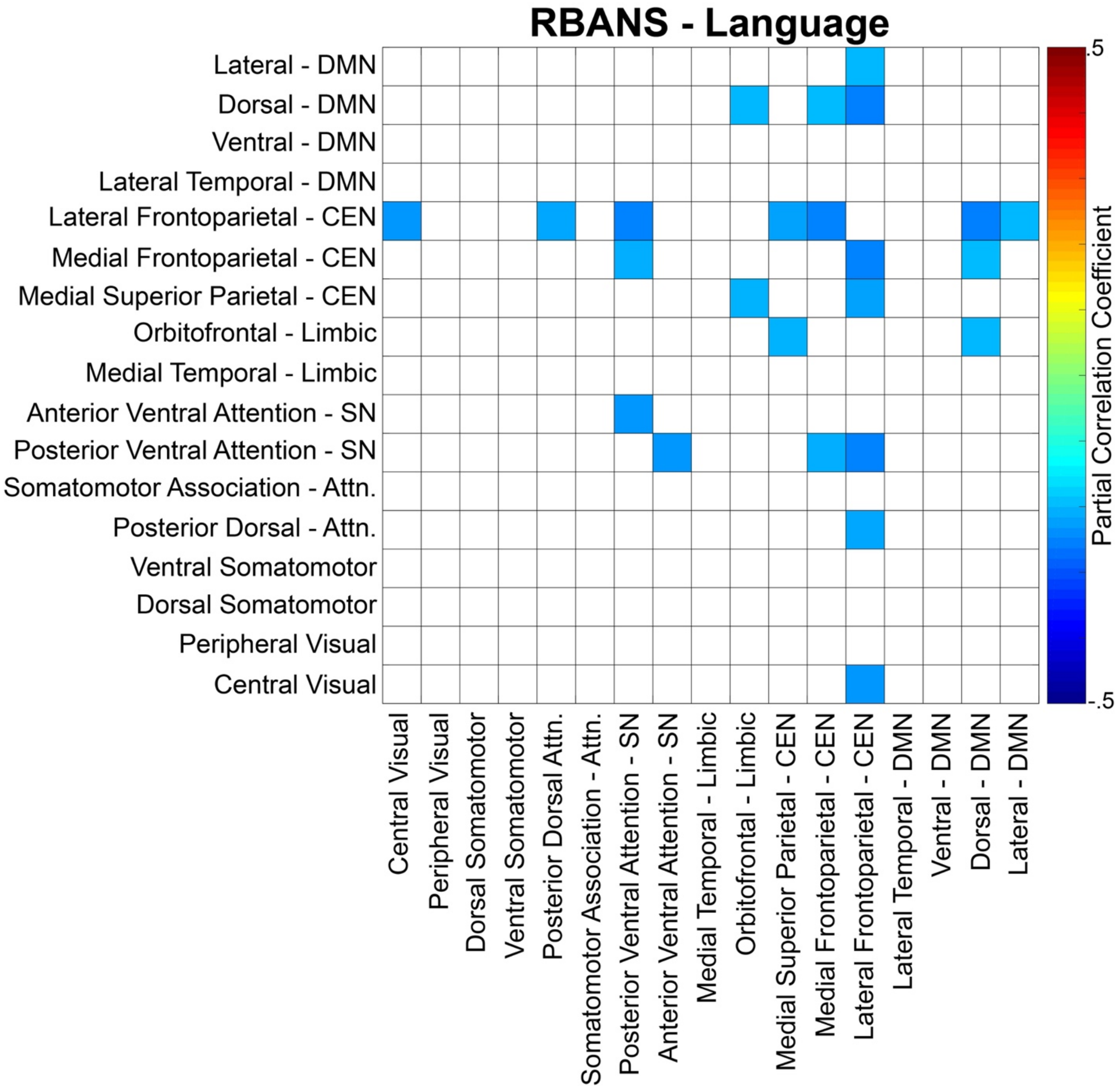

Supplementary Material Figure 4. Correlation Between RBANS Attention Index Scores and Functional Connectivity ($p < .05$, uncorrected).

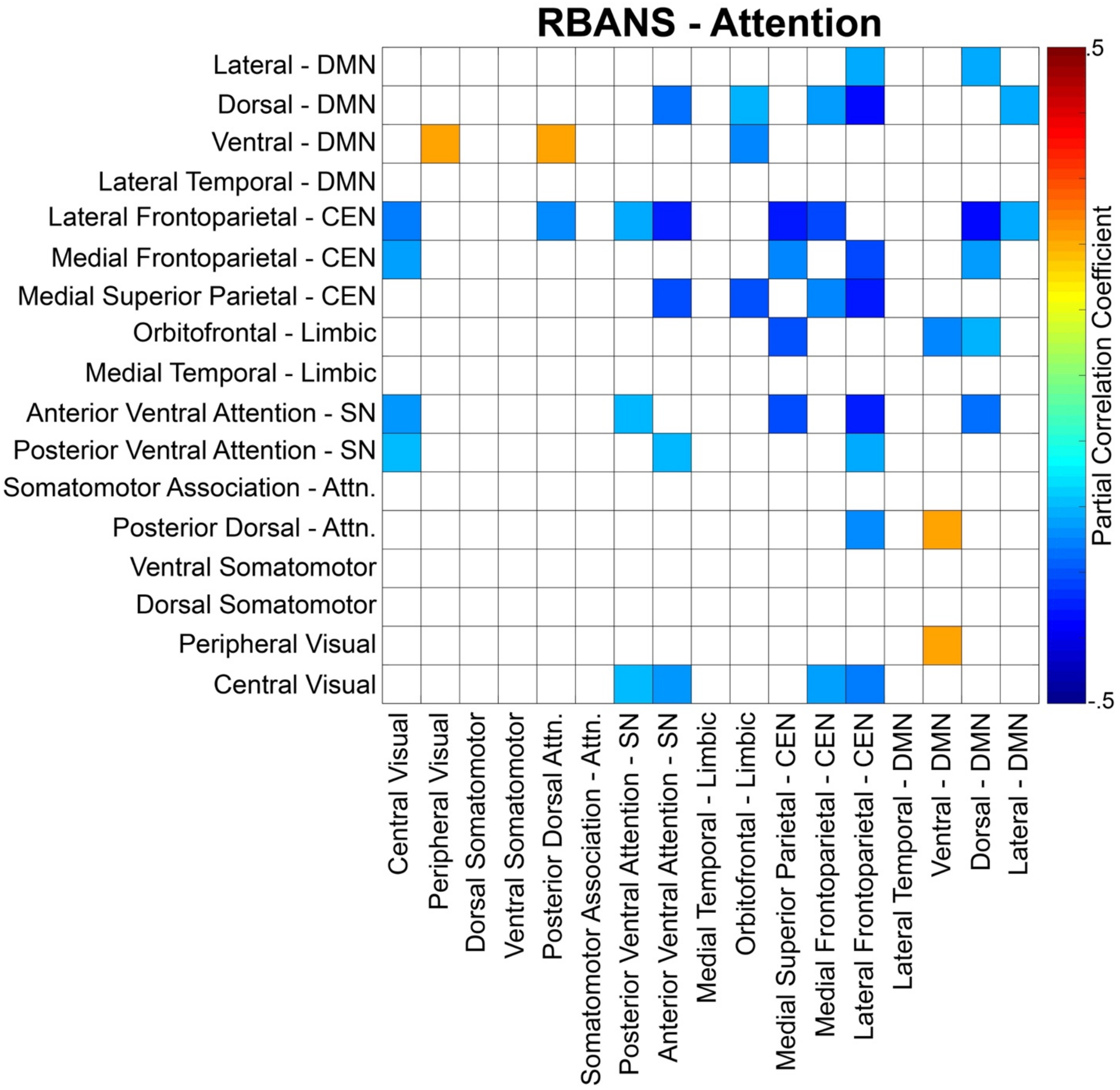

Supplementary Material Figure 5. Correlation Between RBANS Delayed Memory Index Scores and Functional Connectivity ($p < .05$, uncorrected).

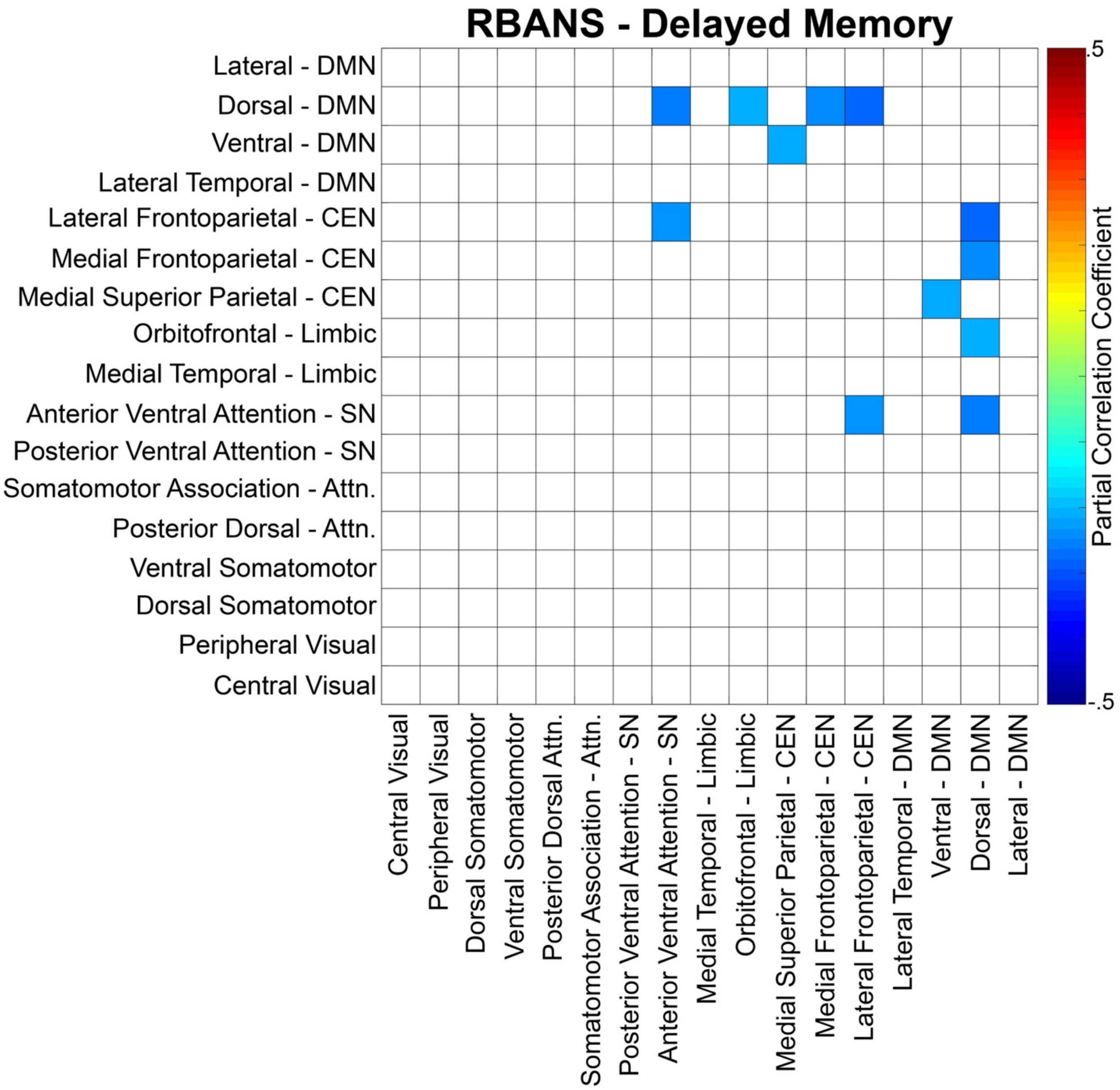

Supplementary Material Figure 6. Correlation Between RBANS Total Scores and Functional Connectivity ($p < .05$, uncorrected).

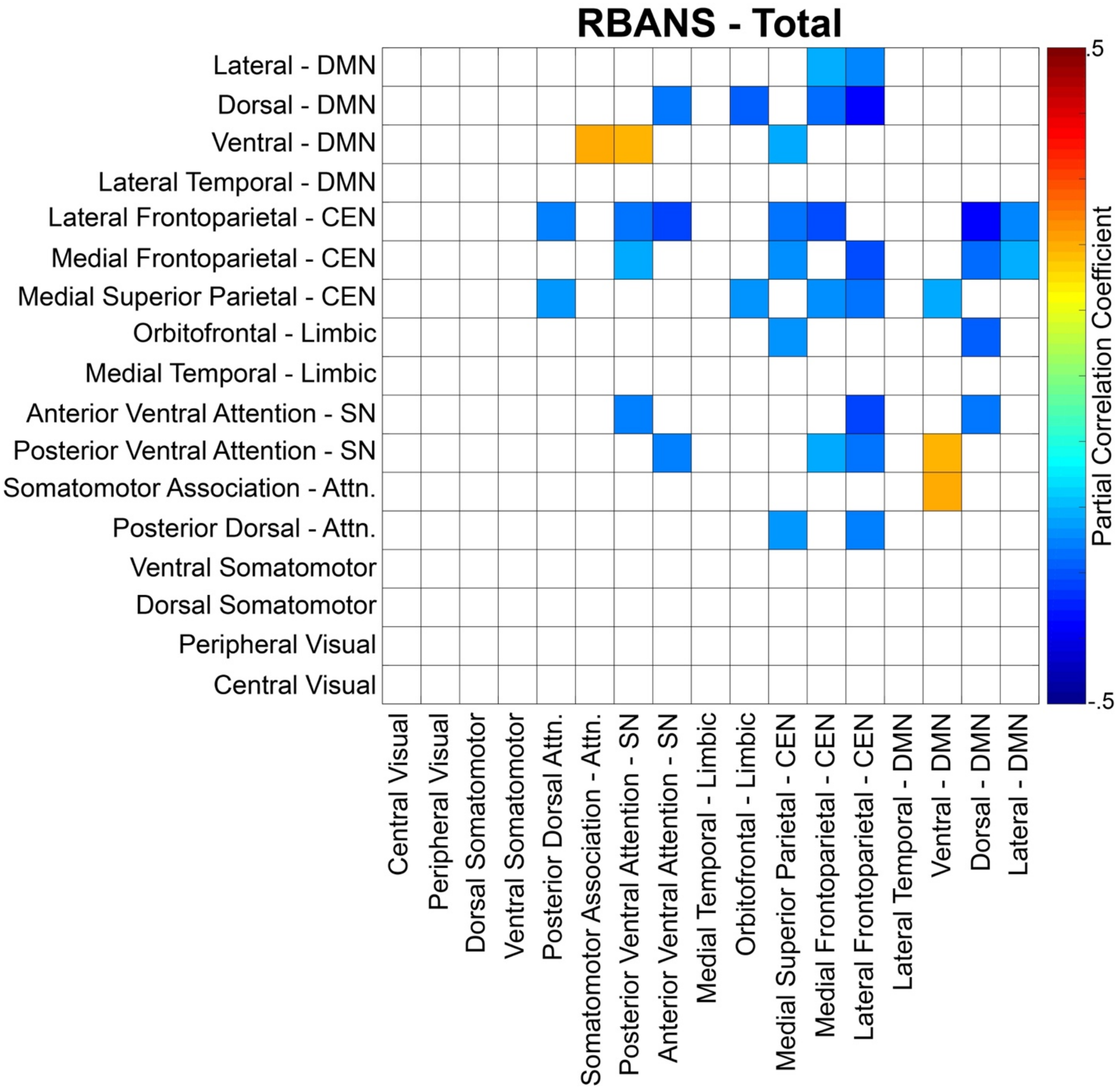

Supplementary Material Figure 7. Functional Connectivity in Alzheimer's Disease Compared to Cognitively Intact Participants ($p < .05$, uncorrected, *$qFDR < .05$).

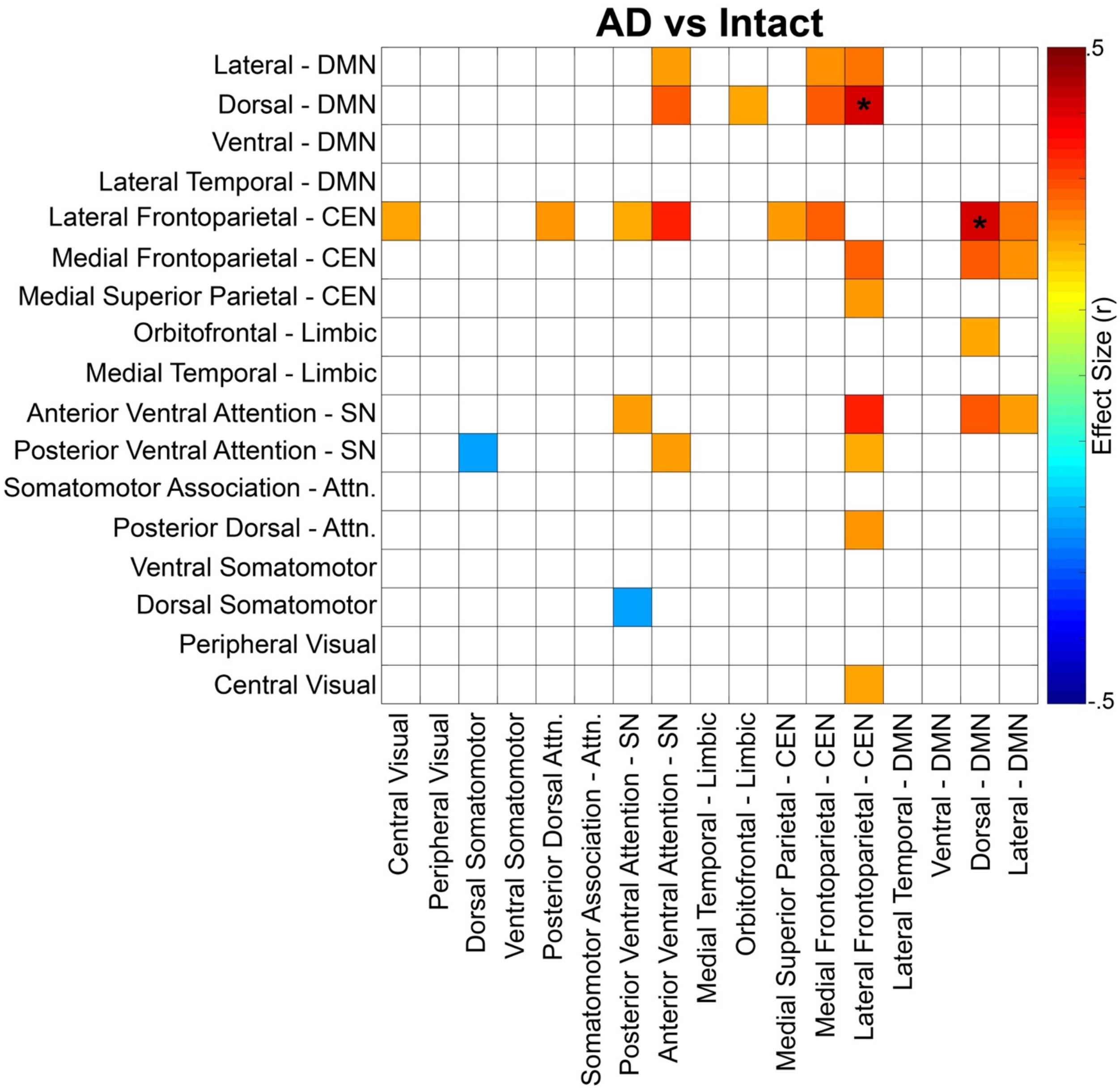

Supplementary Material Figure 8. Functional Connectivity in Alzheimer's Disease Compared to Mild Cognitive Impairment (*p* < .05, uncorrected, **qFDR* < .05).

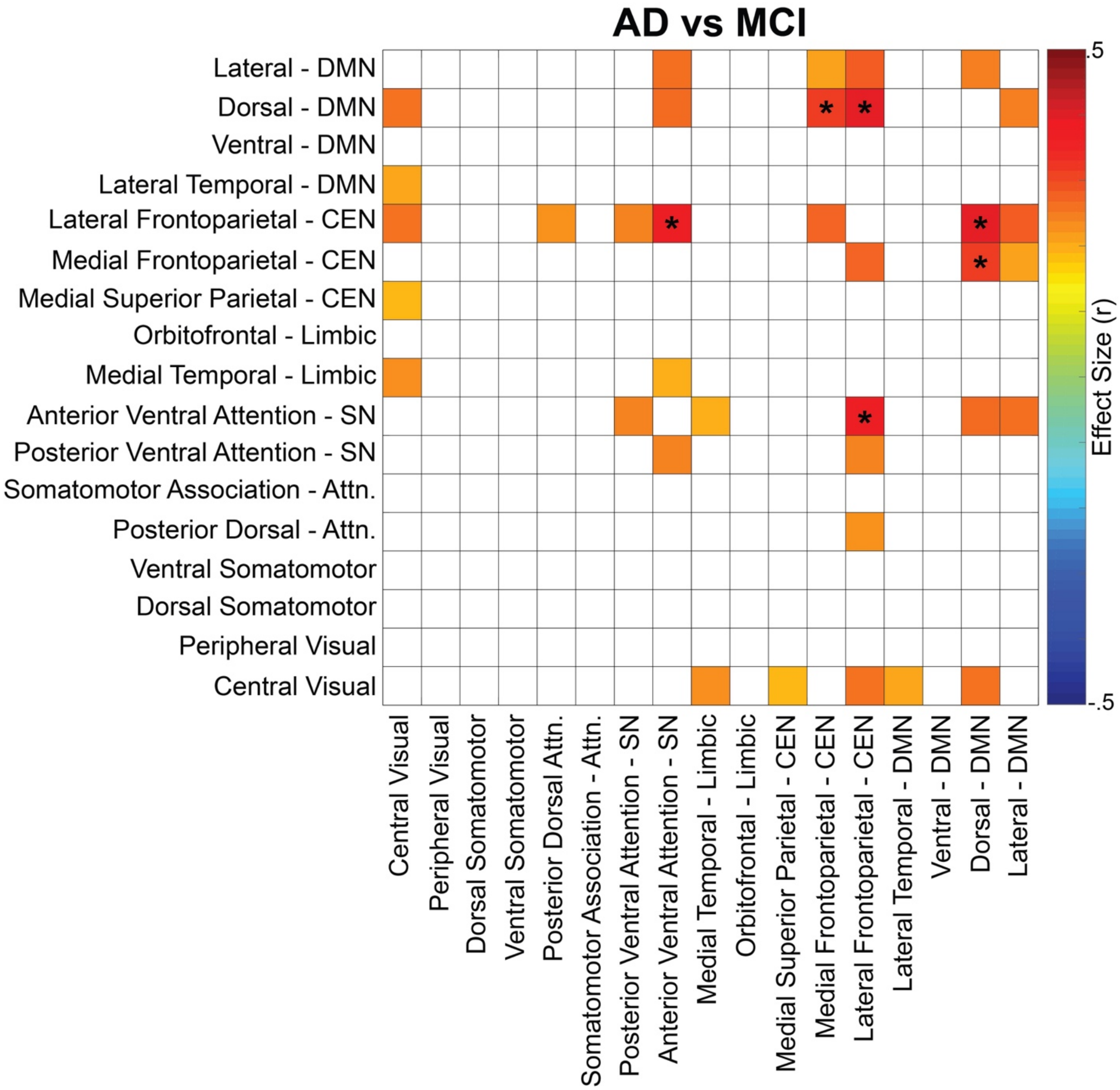

Supplementary Material Figure 9. Functional Connectivity in Mild Cognitive Impairment Compared to Cognitively Intact Participants (p < .05, uncorrected).

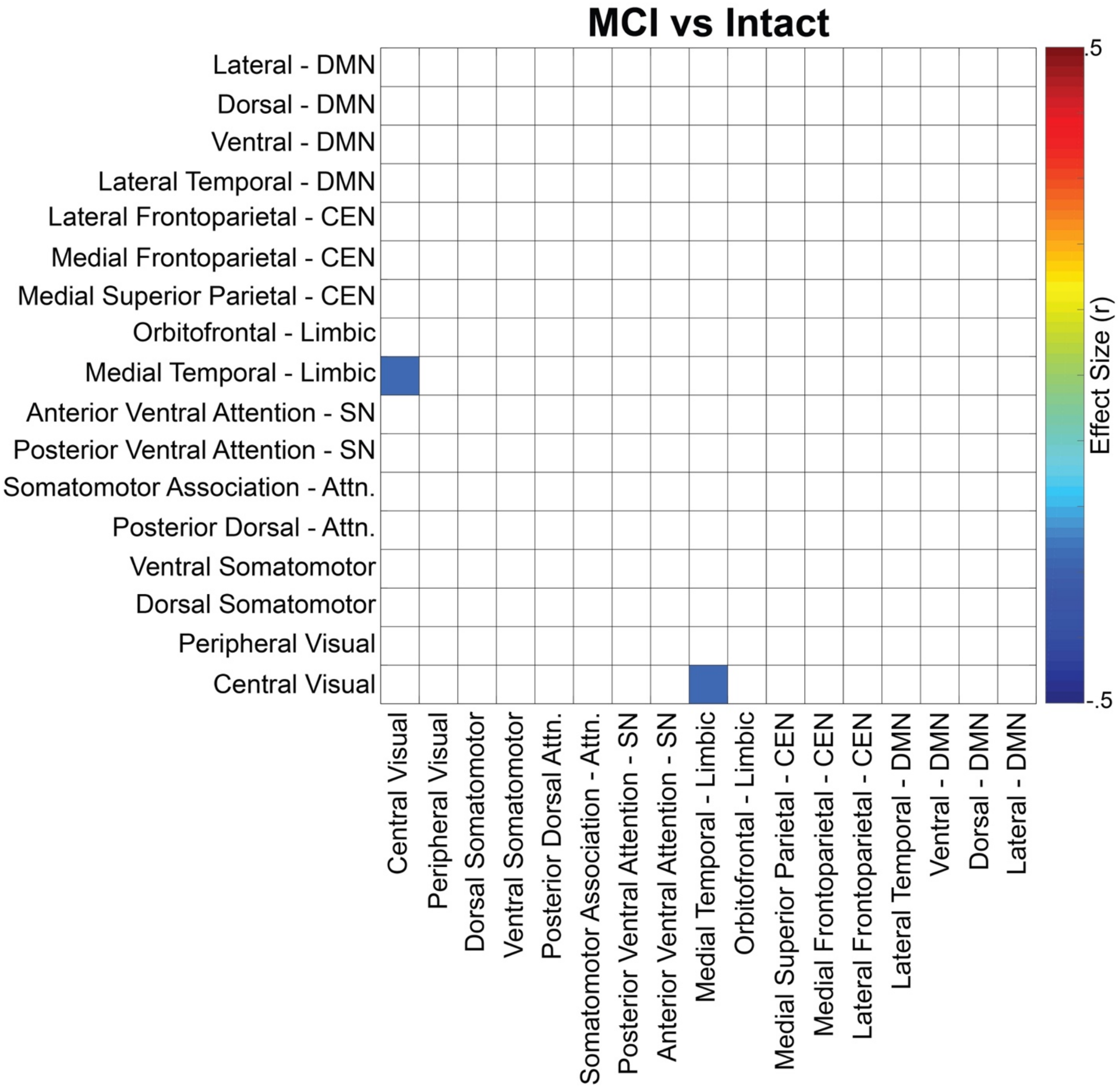

Supplement: King et al. supplementary material 1 — King et al. supplementary material [file S135561772610191Xsup001.pdf]
